# Intraportally delivered stem cell spheroids localize in the liver and protect hepatocytes against GalN/LPS-induced fulminant hepatic toxicity

**DOI:** 10.1186/s13287-019-1337-3

**Published:** 2019-10-16

**Authors:** Shobha Regmi, Shiva Pathak, Tung Pham Thanh, Tiep Tien Nguyen, Jong-Hyuk Sung, Simmyung Yook, Jong Oh. Kim, Chul Soon Yong, Inho Choi, Kyoung-Oh Doh, Pil-Hoon Park, Jun-Beom Park, Yoojin Seo, Bieong-Kil Kim, Dong-Mok Lee, Ik-Jae Moon, Hyung-Sik Kim, Jee-Heon Jeong

**Affiliations:** 1 College of Pharmacy, Yeungnam University, 280 Daehak-ro, Gyeongsan-si, Gyeongbuk-do 38541 Republic of Korea; 2 College of Pharmacy, Yonsei University, Incheon, 21983 Republic of Korea; 3 College of Pharmacy, Keimyung University, Daegu, 42415 Republic of Korea; 4 Department of Medical Biotechnology, Yeungnam University, Gyeongsan, 38541 Republic of Korea; 5 Department of Physiology, College of Medicine, Yeungnam University, Daegu, 42415 Republic of Korea; 6 Department of Periodontics, College of Medicine, The Catholic University of Korea, Seoul, 06591 Republic of Korea; 7 Department of Life Science in Dentistry, School of Dentistry, Pusan National University, Yangsan, 50612 Republic of Korea; 8 Institute for Translational Dental Sciences, Pusan National University, Yangsan, 50612 Republic of Korea; 9 Biomedical Manufacturing Technology Center, Korea Institute of Industrial Technology, Gyeongbuk, 38822 Republic of Korea; 10 WELGENE Inc., Gyeongsan, 38695 Republic of Korea

**Keywords:** Mesenchymal stem cell, 2D-cultured cells, Spheroids, Intraportal delivery, Fulminant hepatic failure

## Abstract

**Background:**

Systemic inflammatory response syndrome (SIRS) is common in severe fulminant hepatic failure (FHF) and has a high mortality rate (20–50%) due to irreversible cerebral edema or sepsis. Stem cell-based treatment has emerged as a promising alternative therapeutic strategy to prolong the survival of patients suffering from FHF via the inhibition of SIRS due to their immunomodulatory effects.

**Methods:**

3D spheroids of adipose-derived mesenchymal stem cells (3D-ADSC) were prepared by the hanging drop method. The efficacy of the 3D-ADSC to rescue FHF was evaluated in a d-galactosamine/lipopolysaccharide (GalN/LPS)-induced mouse model of FHF via intraportal transplantation of the spheroids.

**Results:**

Intraportally delivered 3D-ADSC better engrafted and localized into the damaged livers compared to 2D-cultured adipose-derived mesenchymal stem cells (2D-ADSC). Transplantation of 3D-ADSC rescued 50% of mice from FHF-induced lethality, whereas only 20% of mice survived when 2D-ADSC were transplanted. The improved transplantation outcomes correlated with the enhanced immunomodulatory effect of 3D-ADSC in the liver microenvironment.

**Conclusion:**

The study shows that the transplantation of optimized 3D-ADSC can efficiently ameliorate GalN/LPS-induced FHF due to improved viability, resistance to exogenous ROS, and enhanced immunomodulatory effects of 3D-ADSC.

**Electronic supplementary material:**

The online version of this article (10.1186/s13287-019-1337-3) contains supplementary material, which is available to authorized users.

## Background

Toxins, drugs, viral infections, and genetic and metabolic disorders cause liver dysfunction requiring medical intervention [1]. During fulminant hepatic failure (FHF; also known as acute liver failure (ALF)), bacterial toxins or other hepatotoxins cause massive hepatocellular damage [2, 3]. In addition, systemic inflammatory response syndrome (SIRS) mediated by massive stimulation of innate and/or adaptive immune system is frequently observed in severe cases of FHF. In fact, SIRS is the leading cause of fatality in FHF and 20–50% of FHF patients succumb to irreversible cerebral edema or sepsis with multiple organ dysfunction [4]. Thus, suppression of excessive immune responses during liver dysfunction may reduce the risk of hepatic failure-induced mortality.

Liver transplantation is the only life-saving treatment for permanent liver failure; however, various obstacles, such as limited donor issue, risk of fungal infection during the procedures, immune rejection response against the graft, and long-term immunosuppressant-associated complications decrease the success rate [5, 6]. To overcome the potential risks of whole organ transplantation, non-invasive cell-based therapy using primary hepatocytes is of keen interest for the treatment of liver diseases [7, 8]. However, many aspects of hepatocyte culture, including their isolation, expansion, and preservation, have proven to be difficult, and poor engraftment efficacy limits the hepatocyte transplantation [9, 10]. In this context, mesenchymal stem cells (MSC) are promising candidates for cell therapy because of their multipotent differentiation ability and immunomodulatory activity [11–13]. Furthermore, MSC have been reported to rescue impaired hepatocytes by differentiating into hepatocyte-like cells and/or by improving the inflammatory, fibrotic microenvironment through paracrine effects [14] and preventing further liver damage during FHF [15]. Notably, earlier MSC-based therapies were mainly dependent on the multi-lineage differentiation ability, whereas recent studies have focused on the paracrine and autocrine functions of MSC-derived therapeutic factors [16–18]. Previous reports have revealed that the aggregation of MSC into 3D spheroids causes enhancement in the secretion of paracrine factors and proangiogenic cytokines [19, 20] and protection of the stemness [21, 22]. Furthermore, 3D culture methods have been reported to enhance stem cell survival in vitro as well as in vivo and provide better engraftment efficiency [23, 24]. Thus, 3D culture methods offer a means of improving the efficacy of stem cell therapies and eliminate the need for genetic modification and growth factor delivery.

In cell therapy, the route of cell delivery must be considered deliberately to maximize therapeutic efficacy. In most cases, MSC are administrated via intravenous injection [25–27], which results in non-specific distributions of cells. The cells are primarily accumulated in the lungs, and only a small number of cells localize into the liver despite their homing ability [28]. Although various injection routes have been considered, including delivery into targeted areas, no report has been issued on maximizing the localization of the transplanted cells in models of liver injury. In the current study, we aimed to evaluate the therapeutic potential of adipose-derived mesenchymal stem cells (ADSC) in a d-galactosamine (GalN)/lipopolysaccharide (LPS)-induced murine model of hepatic injury, because it closely resembles FHF [2, 3]. To enhance ADSC engraftment and localization in the liver, we delivered the 3D spheroids derived from ADSC (3D-ADSC) via the portal vein. The liver-entrapped 3D-ADSC helped in the modulation of inflammatory hepatic microenvironment through the secretion of anti-inflammatory cytokines/growth factors and improved outcomes of mesenchymal stem cell therapy in FHF.

## Material and methods

### Cells

Human adipose-derived mesenchymal stem cells (ADSC) were purchased from Zenbio (Morrisville, NC, USA). ADSC were cultured in alpha-MEM (Hyclone, South Logan, UT, USA) containing 10% *v*/*v* fetal bovine serum (Hyclone, South Logan, UT, USA) and 1% *v*/*v* antibiotics/antimycotics (GenDEPOT, Barker, TX, USA). Cells from passages 4–8 were used in this study.

### Preparation and optimization of 3D-ADSC

A previously described hanging drop technique was used to prepare 3D-ADSC [29]. The differently sized spheroids, i.e., 3D-ADSC_500_, 3D-ADSC_1000_, 3D-ADSC_2000_, and 3D-ADSC_4000_ containing 500, 1000, 2000, and 4000 ADSC per spheroid, respectively, were prepared. The viability and size of 3D-ADSC were considered for the optimization of size of the 3D-ADSC for in vivo studies. The viability of differently sized 3D-ADSC was assessed using a live/dead assay kit (Molecular Probes, Eugene, OR, USA), and Western blot analysis as reported previously [30].

### Induction of FHF

Balb/c nude mice (7–8 weeks old) purchased from Orient Biosciences (Seoul, Republic of Korea) were used in this study. Animals were housed in the animal facility of Yeungnam University. Animal experimental protocols were approved by the regulations of Institutional Animal Care and Use Committee (IACUC) of Yeungnam University (IACUC: YL 2018-028). For the determination of the optimal dose required to induce FHF, mixtures of GalN (Carbosynth, Old Station Business Pk, Compton RG20 6NE, UK) (1000, 1500, 2000, or 3000 mg/kg) and LPS (Sigma, St. Louis, MO, USA) (20 μg/kg) were administered intraperitoneally (i.p.). The anti-inflammatory effect of stem cells was evaluated using an optimized GalN/LPS dose (single injection; 1500 mg/kg GalN and 20 μg/kg LPS). Mice were divided randomly into different groups after the induction of ALF.

### Intraportal delivery of ADSC

The therapeutic efficacy of ADSC was determined by infusing cells via the portal vein 5 h after GalN/LPS injection. One thousand 3D-ADSC_1000_ or 1 × 10^6^ 2D-ADSC were suspended in 100 μL PBS and injected into the portal vein using an insulin syringe. Sham group received an intraportal injection of 100 μL PBS. Subsequently, mice were monitored 6 hourly and blood samples were obtained at 1, 3, 5, and 7 days after transplantation. Serum glutamate-pyruvate transaminase (GPT) and serum glutamic oxaloacetic transaminase (GOT) levels were measured via the colorimetric method using a chemistry analyzer (DRI-CHEM 4000i, FUJIFILM Corporation, Tokyo, Japan).

### DNA extraction and real-time PCR

At 10 h after MSC transplantation, the livers, lungs, and hearts were isolated to investigate the presence of the transplanted cells in these organs. Depending on the weight of the organ, lysis buffer was added and homogenized. Afterwards, DNA was extracted using a genomic DNA extraction kit (Bioneer Corp, Daejeon, South Korea) and quantified using a spectrophotometer (Nanodrop; TECAN). Real-time PCR (RT-PCR) was performed using 25 ng DNA, 5 μL SYBER green (Thermo Scientific, Waltham, MA, USA), 37.5 nM ALU primer, and 450 nM GAPDH primer. The primer sequences used were as follows: ALU, 5′-GTCAGGAGATCGAGACCATCCC-3′ (F) and 5′-TCCTGCCTCAGCCTCCCAAG-3′ (R), and GAPDH, 5′-ACCACAGTCCATGCCATCAC-3′ (F) and 5′-TCCACCACCCTGTTGCTGTA-3′ (R). Calibration curve was prepared using crossing point (Cp) values obtained with various amounts of human DNA (16%, 8%, 4%, 2%, 1%, 0.5%, 0.25%, 0.125%, 0.0625%, 0.03125%, 0.0156%, 0.0078%, and 0.0039%).

### Levels of ROS and ROS-related enzymes in 3D-ADSC_1000_

To understand the mechanism responsible for the effects of 3D-ADSC_1000_ transplantation, amounts of ROS produced by 2D-ADSC and 3D-ADSC_1000_ were evaluated by the fluorescence method using 20 μM of 2′,7′-dichlorofluorescein diacetate (DCFDA) (Sigma-Aldrich, St. Louis, MO, USA) and normalized with the DNA contents measured using PicoGreen Kit (Molecular Probes, Eugene, OR, USA). Similarly, levels of the antioxidant enzymes superoxide dismutase 2 (SOD2), catalase (CAT), and hemeoxygenase-1 (HO-1) were determined by the Western blot analysis using antibodies against SOD2 (Cell Signaling Technology, Danvers, MA, USA), CAT (Cell Signaling), and HO-1 (Cell Signaling).

### Viability of 3D-ADSC_1000_ under oxidative stress condition

In order to examine cell viability in a highly oxidative environment that resembles the FHF liver, 3 × 10^4^ monolayer-cultured MSCs or 30 spheroids (each spheroid containing 1000 MSCs) were cultured with different concentrations of TBHP (tert-butyl hydroperoxide) (Tokyo Chemical Industry, Nihonbashi-honcho, Chuo-ku, Tokyo, Japan) (200 and 400 μM) for 24 h in a 96-well plate. Viability was assayed by using live/dead imaging and Cell Counting Kit-8 (CCK-8) as described previously [30].

### Anti-inflammatory effects of 3D-ADSC_1000_ and 2D-ADSC

The levels of prostaglandin E2 (PGE_2_) secreted in supernatants after 3 days of culture of 2D-ADSC and 3D-ADSC_1000_ were measured using a PGE_2_ ELISA kit (R & D Systems, Minneapolis, MN, USA). Similarly, in vitro anti-inflammatory effects of 3D-ADSC_1000_ and 2D-ADSC were assessed by culturing them with activated macrophages. Briefly, RAW 264.7 macrophages were cultured at a density of 4 × 10^4^ per well in a 96-well plate for 24 h and activated by adding 1 mg/mL of LPS for 90 min. Ten 3D-ADSC_1000_ or 1 × 10^4^ 2D-ADSC were added to each well, and 10 h later, supernatants were collected, centrifuged, and stored at − 70 °C until analysis. Levels of IL-10 and TNF-α were measured by ELISA using a Mouse IL-10 ELISA kit (R & D Systems, Minneapolis, MN, USA) or a Mouse TNF-α kit (Elabscience, Bethesda, MD, USA), respectively. Finally, levels of ROS in activated macrophages were measured by the fluorescence method using DCFDA.

### In vivo assessment of the anti-inflammatory effect of 3D-ADSC_1000_ and 2D-ADSC

For the in vivo assessment of the effect of 3D-ADSC_1000_ and 2D-ADSC in attenuating inflammation in the FHF model, polarization of macrophage in the liver was evaluated using flow cytometry. After 7 h of cell transplantation, isolation of monocytes from the liver was conducted as described previously [31]. The isolated cells were stained with anti-mouse PE-Cy7-conjugated CD11b (Biolegend, San Diego, CA, USA), PE-conjugated F4/80 (Biolegend), APC-Cy7-conjugated CD86 (Biolegend), and PE-Cy7-conjugated CD206 (Biolegend) and analyzed by BD FACS Verse flow cytometer (BD Biosciences, San Jose, CA, USA). To check M1 and M2 phenotypes of macrophage, F4/80^+^ population was selected, and the expression of CD86 (M1 surface marker) and CD206 (M2 surface marker) was evaluated. Median fluorescence intensity (MFI) was calculated using FlowJo Software (FlowJo LLC, OR, USA). In addition, the concentration of IL-10 in serum was also evaluated by ELISA.

### Histochemical analysis

3D-ADSC_1000_- and 2D-ADSC-injected mice were sacrificed 7 h after injections, and the livers were surgically removed, fixed in 4% paraformaldehyde solution, and embedded in paraffin. Samples were then sectioned at 5 μm, deparaffinized in xylene, rehydrated using an alcohol series, and stained with hematoxylin and eosin (H&E).

### Ex vivo imaging

Cells were stained with VivoTrack680 (pernElmer, Boston, MA, USA) according to the manufacturer’s protocol. Then, 1 × 10^6^ monolayer cells or 1000 3D-ADSC_1000_ were injected into the portal vein via a surgical process. At 7 h post-transplantation, different organs were isolated to observe the fluorescence using in vivo imaging system (FOBI; NeoScience, Suwon, Korea).

### Statistical analysis

Results are expressed as mean ± SEM. Data were analyzed using GraphPad Prism software version 5 (GraphPad Software, La Jolla, CA, USA). Unless otherwise stated, unpaired *t* test was performed to calculate the statistical significance values. Statistical significance was accepted for *p* < 0.05.

## Results

### Optimization of 3D-ADSC

**Fig. 1:**
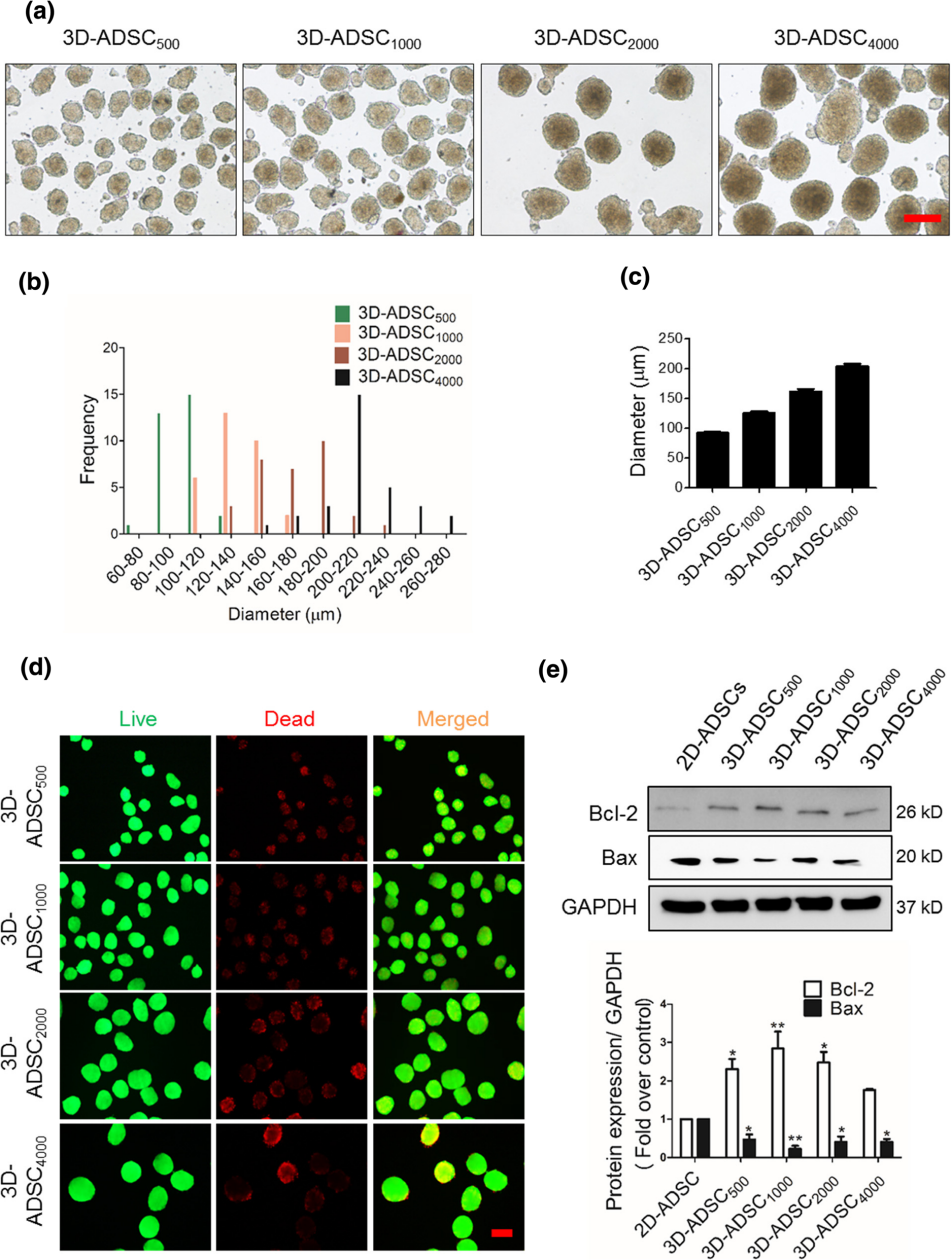
Formation of 3D spheroids of different sizes. **a** Morphology of 3D-ADSC, magnification: × 100, scale bar: 200 μm. **b** Size distribution. **c** Size of 3D-ADSC (*n* = 31) where values represent mean ± SEM. **d** Live/dead images for the determination of viability of differently-sized 3D-ADSC, magnification: × 100, scale bar: 200 μm. **e** Representative blot showing cell viability of 2D-ADSC and differently-sized 3D-ADSC and quantitative expressions of proteins measured using GelQuantNET software. The values represent mean ± SEM of three independent experiments. 3D-ADSC_500_, 500 ADSC per spheroid; 3D-ADSC_1000_, 1000 ADSC per spheroid; 3D-ADSC_2000_, 2000 ADSC per spheroid; 3D-ADSC_4000_, 4000 ADSC per spheroid. *p < 0.05 and **p < 0.01

To optimize the size of spheroids, we prepared 3D-ADSC of different sizes and compared cell viability with 2D-ADSC. The average diameters of 3D-ADSC_500_, 3D-ADSC_1000_, 3D-ADSC_2000_, and 3D-ADSC_4000_ were 91.97 ± 11.80, 125.00 ± 15.93, 161.70 ± 24.39, and 203.86 ± 25.05 μm, respectively (Fig. 1a–c). Interestingly, Western blot analysis revealed two- to threefold increase in expressions of Bcl-2 (anti-apoptotic) and two- to threefold decrease in expressions of Bax (pro-apoptotic) in 3D-ADSC with different cell numbers when compared to the 2D-ADSC. Among the 3D-ADSC groups containing different numbers of cells, 3D-ADSC_1000_ was chosen for further experiments because this group exhibited higher cell viability and anti-apoptotic property compared to the other spheroid groups (Fig. 1d, e). In addition, given that spheroids with size exceeding 150 μm suffer from hypoxia-induced cell death after transplantation [32], 3D-ADSC_1000_ with size ~ 125 μm was expected to have improved efficacy, as well as minimum risk of thrombosis and embolism after transplantation.

### Intraportal delivery of 3D-ADSC_1000_ ameliorated FHF

**Fig. 2:**
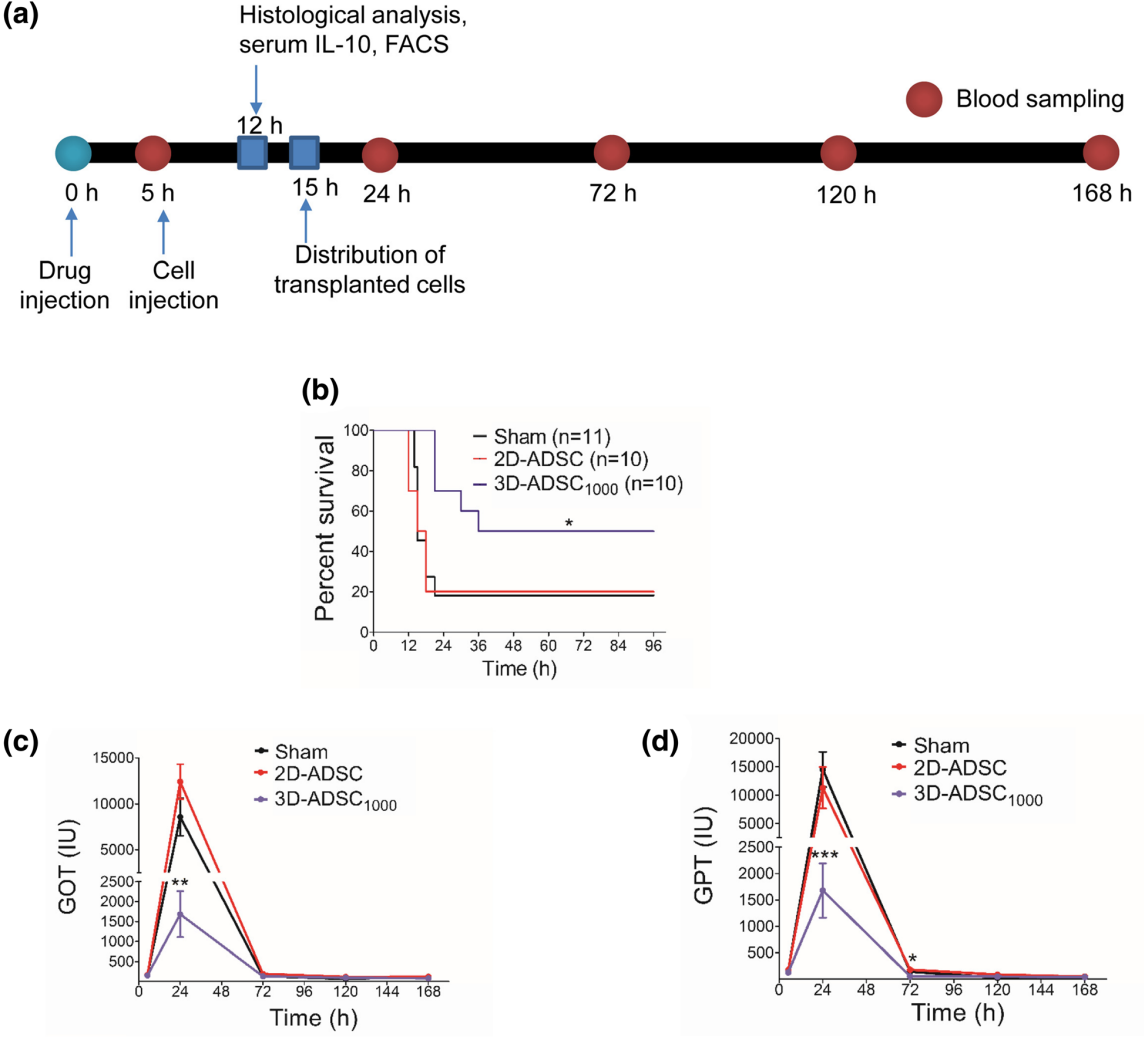
3D-ADSC_1000_ rescued mice from GalN/LPS-induced lethal FHF. **a** Timeline of the study. **b** Survival curve of 1500 mg/kg GalN and 20 μg/kg LPS-treated BALB/c nude mice injected with PBS, 1 × 10^6^ 2D-ADSC, or 1000 3D-ADSC_1000_. Log-rank (Mantel-Cox) test was used to calculate the statistical significance. **c** Serum glutamic oxaloacetic transaminase (GOT) levels. **d** Serum glutamic pyruvic transaminase (GPT) levels. GPT and GOT were analyzed in the surviving mice via the colorimetric method using a chemistry analyzer. Data represent mean ± SEM. *p < 0.05, **p < 0.01, and ***p < 0.001

Dosage of GalN and LPS was optimized, and 1500 mg/kg GalN and 20 μg/kg LPS were chosen for the induction of FHF in BALB/c nude mice, which resulted in the deaths of 2 out of 3 mice 24 h after administration (Additional file 1: Figure S1). To assess the efficacy of ADSC in FHF, 1 × 10^6^ 2D-ADSC or 1000 3D-ADSC_1000_ were delivered via the portal vein. Figure 2a shows the timeline of the study. Transplantation of 3D-ADSC_1000_ significantly improved the survival of mice (survival rate 50%) compared with the sham-operated group (18%) and the 2D-ADSC-treated group (survival rate 20%) (Fig. 2b).

Serum levels of GOT and GPT in the sham, 2D-ADSC, and 3D-ADSC_1000_ groups 5 h after GaLN/LPS injection, were significantly higher than those of untreated mice (untreated mice; GOT 110 ± 20 and GPT 32 ± 9) (Additional file 1: Figure S2). Notably, no significant difference was observed in GPT and GOT levels among all the experimental groups at the time of transplantation. However, at 19 h after cell administration, the levels of serum GPT and GOT were markedly lower in the 3D-ADSC_1000_-treated group compared to the 2D-ADSC-treated group (GPT: *p* < 0.01, GOT: *p* < 0.001) or the sham group (GPT: *p* < 0.001, GOT: *p* < 0.01), indicating a reduction in the severity of liver injury after spheroid transplantation. Similarly, at day 3 post-transplantation, serum GPT levels in the 3D-ADSC_1000_ group were significantly lower compared to those in the 2D-ADSC (*p* < 0.01) and sham (*p* < 0.05) groups (Fig. 2c, d).

**Fig. 3:**
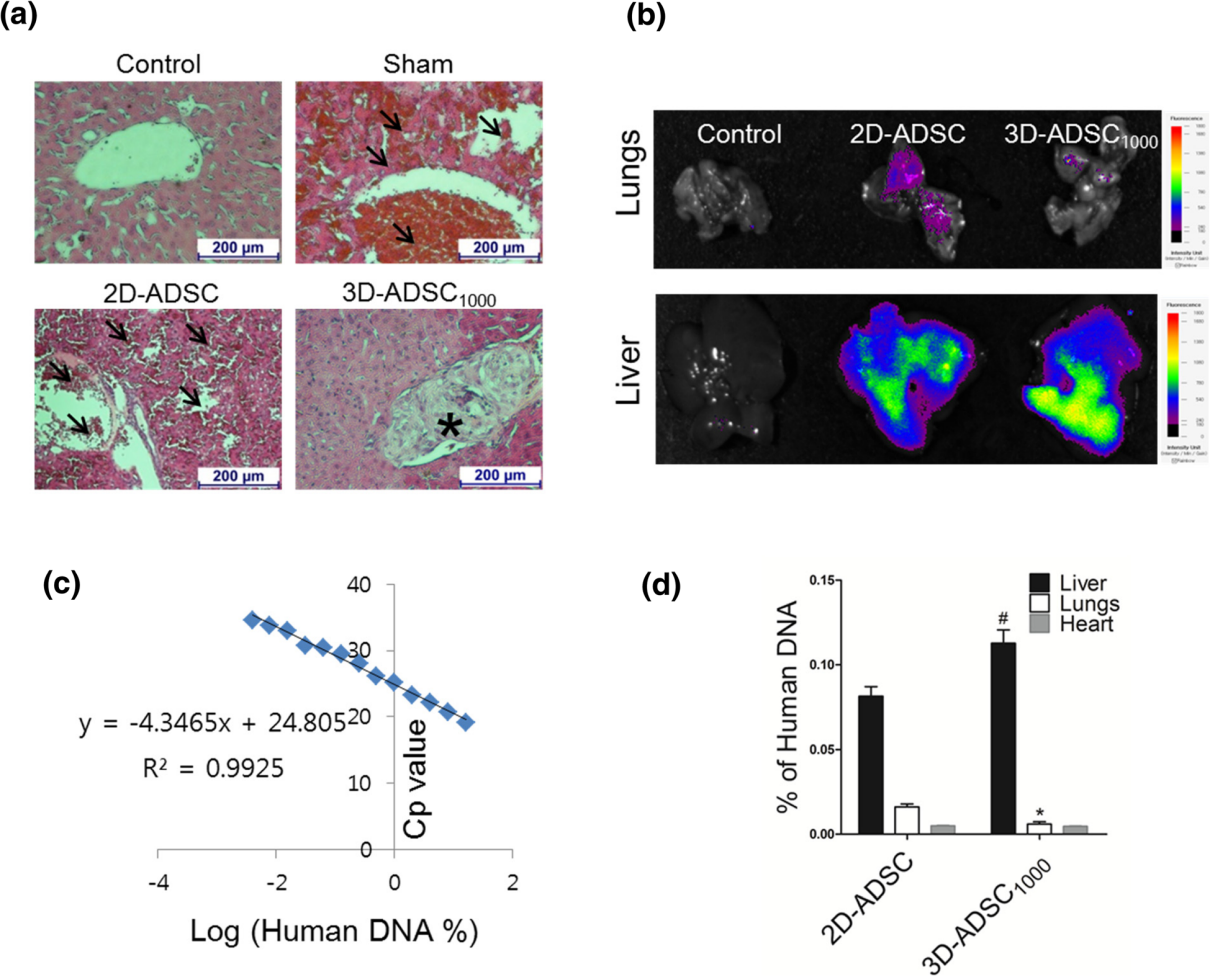
Fate of 2D- and 3D-cultured MSCs after transplantation via portal vein. **a** Histology of the liver 12 h after GalN/LPS administration; magnification: × 100, scale bar: 200 μm. Arrows indicate thrombosis, and the asterisk indicates localization of 3D-ADSC_1000_ in the liver. **b** Ex vivo imaging to observe the extrahepatic distribution of the transplanted cells after intraportal delivery. At 7 h of intraportal delivery of MSCs, ex vivo imaging was performed. Fluorescence was observed in the lungs of the 2D-ADSC-delivered group. However, negligible fluorescence was observed in the lungs of the 3D-ADSC_1000_-delivered group. In addition, significantly higher fluorescence intensity was observed in the liver of the 3D-ADSC_1000_-transplanted group compared to the 2D-ADSC-transplanted group. **c** Calibration curve for the determination of human DNA. **d** Percentage of human DNA observed in vital organs at 10 h of MSC transplantation (*n* = 3). The values represent mean ± SEM. *p < 0.05 vs 2D-ADSC in the lungs and ^#^*p* < 0.05 vs 2D-ADSC in the liver

Histological analysis of liver sections from the sham and 2D-ADSC-treated groups revealed an accumulation of erythrocytes in the extra-sinusoidal region, signifying the damage of hepatic parenchymal and sinusoidal endothelial cells. In addition, the high extent of thrombosis was observed in the liver of 2D-ADSC-transplanted mice. In contrast, minimal thrombosis was observed in the recipients with 3D-ADSC_1000_ (Fig. 3a). Gross observation at 7 h after cell delivery demonstrated the formation of a substantial infarcted region in the liver of the 2D-ADSC group, but no significant infarction was observed in the 3D-ADSC_1000_ group (Additional file 1: Figure S3).

### Intraportally transplanted 3D-ADMSC_1000_ localized in the liver

Histological analysis revealed the localization of transplanted 3D-ADSC_1000_ in the portal triad. In addition, H&E staining showed an intact morphology of 3D-ADSC_1000_ at 7 h of transplantation (Fig. 3a). Similarly, ex vivo imaging of the liver and lungs also demonstrated the localization of ADSC in the liver and minimal migration into the lungs in the 3D-ADSC_1000_-transplanted group (Fig. 3b). Further, to quantify total human ADSC after transplantation, the expression of human-specific ALU sequence was evaluated in the liver, lungs, and heart. A calibration curve was generated using different percentages of human DNA mixed with mouse DNA (Fig. 3c). This equation was further used to calculate the total percent of human DNA in mouse tissue. Results revealed a higher number of cells distributed in the liver of the 3D-ADSC_1000_-transplanted group compared to the 2D-ADSC-transplanted group. In contrast, the percentage of human DNA in the lungs of the 3D-ADSC_1000_-transplanted group was significantly lower than that of the 2D-ADSC-transplanted group, suggesting a minimal migration of ADSC from the spheroids to extrahepatic tissues after transplantation. No significant migration of MSC was observed in the heart of both groups (Fig. 3b, d).

### 3D-ADSC_1000_ were resistant to oxidative stress

**Fig. 4:**
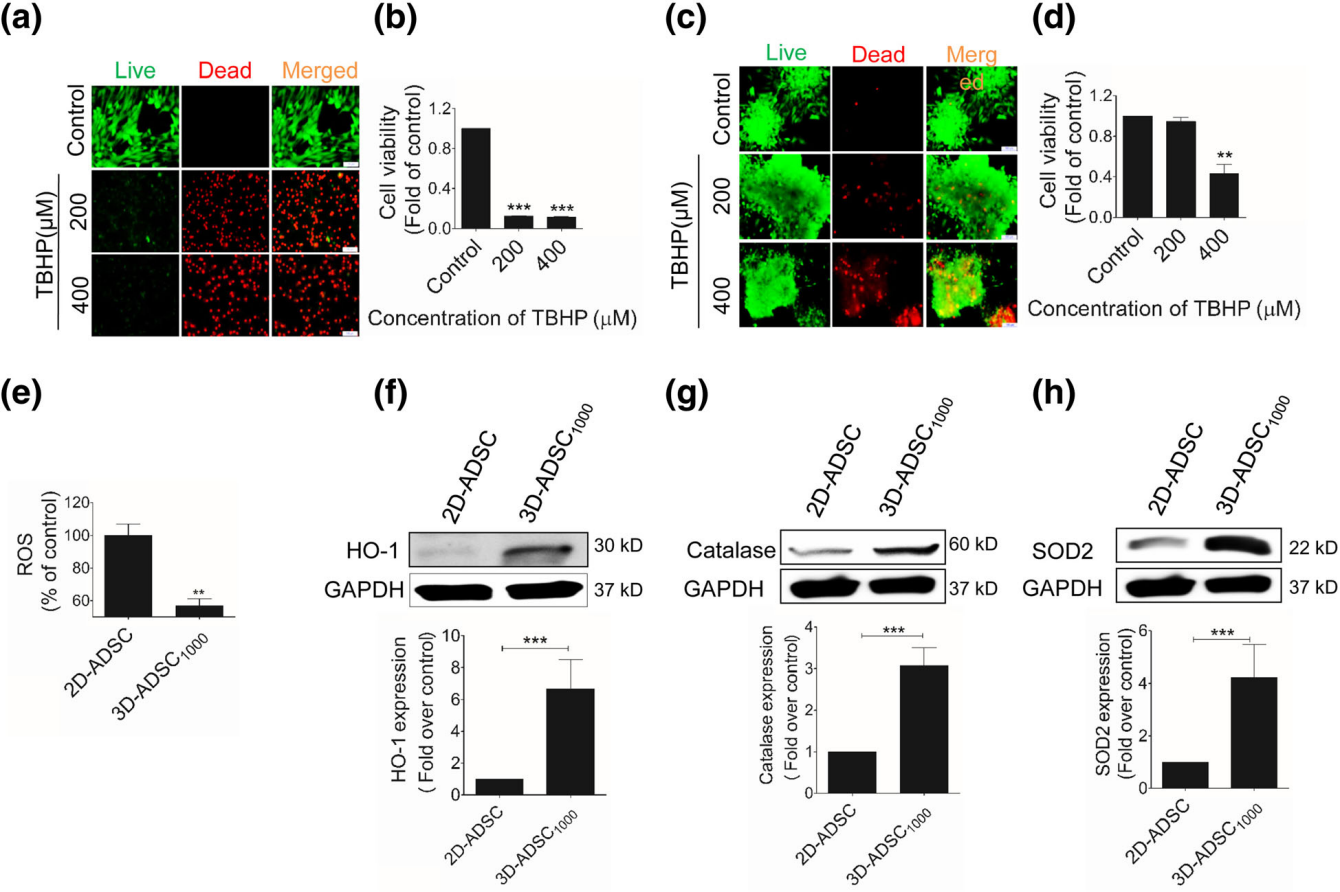
3D-ADSC_1000_ resisted to oxidative stress in vitro. **a** Qualitative analysis of the viability of 2D-ADSC 24 h after treatment with exogenous oxidant (TBHP) using live/dead imaging; magnification × 100, scale bar 100 μm. **b** Quantitative analysis of viability of 2D-ADSC 24 h after treatment with exogenous oxidant (TBHP) using CCK-8 assay. Data represent the mean ± SEM of three independent experiments. **c** Representative live/dead image showing the viability of 3D-ADSC_1000_ 24 h after treatment with TBHP; magnification × 100, scale bar 100 μm. **d** Quantitative assessment of viability of 3D-ADSC_1000_ 24 h after treatment with TBHP using CCK-8 assay. The graph represents mean ± SEM of three independent experiments. **e** Basal levels of ROS in 2D-ADSC and 3D-ADSC_1000_ determined by using DCFDA reagent (mean ± SEM, three independent experiments). **f** Representative blot for basal level expressions of HO-1 in 3D-ADSC and 2D-ADSC and quantitative evaluation of the HO-1 expression from three independent experiments using GelQuantNET software. Representative blots and quantitative expressions of the antioxidant enzymes **g** SOD2 and **h** catalase (mean ± SEM, three independent experiments). **p < 0.01 and ***p < 0.001

To evaluate the effect of oxidative stress on the viability of 2D-ADSC and the spheroids in vitro, 2D-ADSC and 3D-ADSC_1000_ were treated with TBHP. Results unveiled a higher number of cells stained with ethidium homodimer-1 in TBHP-treated 2D-ADSC (Fig. 4a) and < 20% of cells were viable at both concentrations (Fig. 4b). In contrast, cell viability in the 3D-ADSC_1000_ was not diminished by TBHP at 200 μM and approximately 40% of the cells remained viable when they were treated with TBHP at 400 μM (Fig. 4c, d). To investigate the mechanism underlying the enhanced resistance of 3D-ADSC_1000_ against oxidative stress, we measured levels of ROS, HO-1, SOD2, and CAT in 2D-ADSC and 3D-ADSC_1000_. Interestingly, we found a significantly lower (~ 50% lower) level of ROS in 3D-ADSC_1000_ compared to 2D-ADSC (Fig. 4e). Similarly, the expression of antioxidant proteins including HO-1, CAT, and SOD2 were remarkably higher in 3D-ADSC_1000_ compared to those of 2D-ADSC (Fig. 4f–h). These findings suggest that 3D-ADSC_1000_ possess improved resistant mechanism against oxidative stress through the upregulation of anti-oxidative enzymes.

### 3D-ADSC_1000_ transplantation exerted improved immunomodulation

**Fig. 5:**
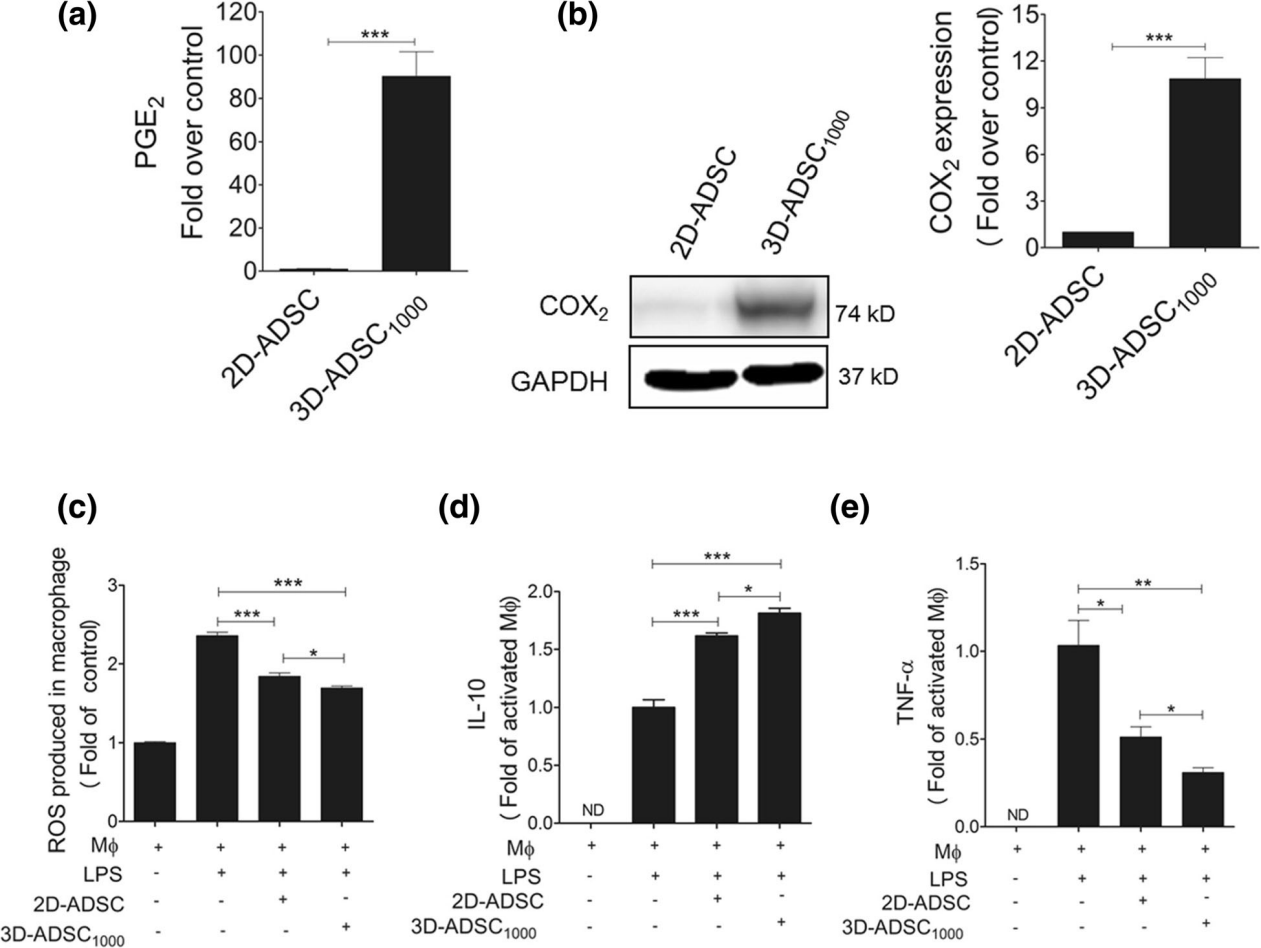
3D-ADSC_1000_ exerted enhanced immunomodulatory activity. **a** Basal levels of PGE_2_ secreted by 2D-ADSC and 3D-ADSC_1000_. Data represent mean ± SEM of three independent experiments. **b** Representative blot of COX_2_ expression in 2D-ADSC and 3D-ADSC_1000_ and quantitative estimation of COX_2_ expression using GelQuantNET software. Data represent mean ± SEM from three independent experiments. **c** Evaluation of ROS production in LPS-activated macrophages after co-culture with 2D-ADSC or 3D-ADSC_1000_ using fluorescence method. The graph represents mean ± SEM from three independent experiments (one-way ANOVA). **d** IL-10 (anti-inflammatory) secretions after co-culturing activated macrophages with 2D-ADSC or 3D-ADSC_1000_ for 10 h using ELISA. Data indicate mean ± SEM from three independent experiments (one-way ANOVA). **e** TNF-α secretion after co-culture of activated macrophage with 2D-ADSC or 3D-ADSC_1000_ for 10 h. Data indicate mean ± SEM from three independent experiments. *p < 0.05, **p < 0.01, and ***p < 0.001

The GalN/LPS-induced FHF model is known to be accompanied by the activation of Kupffer cells and monocytes, which lead to inflammatory reactions in the liver, hepatocyte death, and systemic inflammation [33]. As PGE_2_ has been reported to play a vital role in the immunosuppressive effect of mesenchymal stem cells on monocytes, we quantified amounts of PGE_2_ secreted by 2D-ADSC and 3D-ADSC_1000_. Interestingly, we found a dramatically higher level of PGE_2_ secreted by 3D-ADSC_1000_ (~ 90-fold) compared to that of 2D-ADSC (Fig. 5a). Furthermore, COX_2_, which regulates PGE_2_ secretion, was markedly upregulated in 3D-ADSC_1000_ as compared with 2D-ADSC (Fig. 5b). Interestingly, when 2D-ADSC or 3D-ADSC_1000_ were separately co-cultured with the activated macrophages, ROS production in the macrophages co-cultured with 3D-ADSC_1000_ was remarkably attenuated as compared to that in the macrophages co-cultured with 2D-ADSC (*p <* 0.05) (Fig. 5c). In addition, production of IL-10 was significantly elevated when the macrophages were co-cultured with the 2D-ADSC or 3D-ADSC_1000_ compared to the LPS-stimulated macrophages (*p* < 0.0001). Notably, IL-10 secretion was greater in the macrophages co-cultured with 3D-ADSC_1000_ than in the macrophages co-cultured with 2D-ADSC (Fig. 5d). In contrast, TNF-α secretion was reduced when the activated macrophages were co-cultured with 2D-ADSC or 3D-ADSC_1000_, but this inhibitory effect was significantly higher when the macrophages were co-cultured with 3D-ADSC_1000_ (*p* < 0.01 vs 2D-ADSC) (Fig. 5e).

**Fig. 6:**
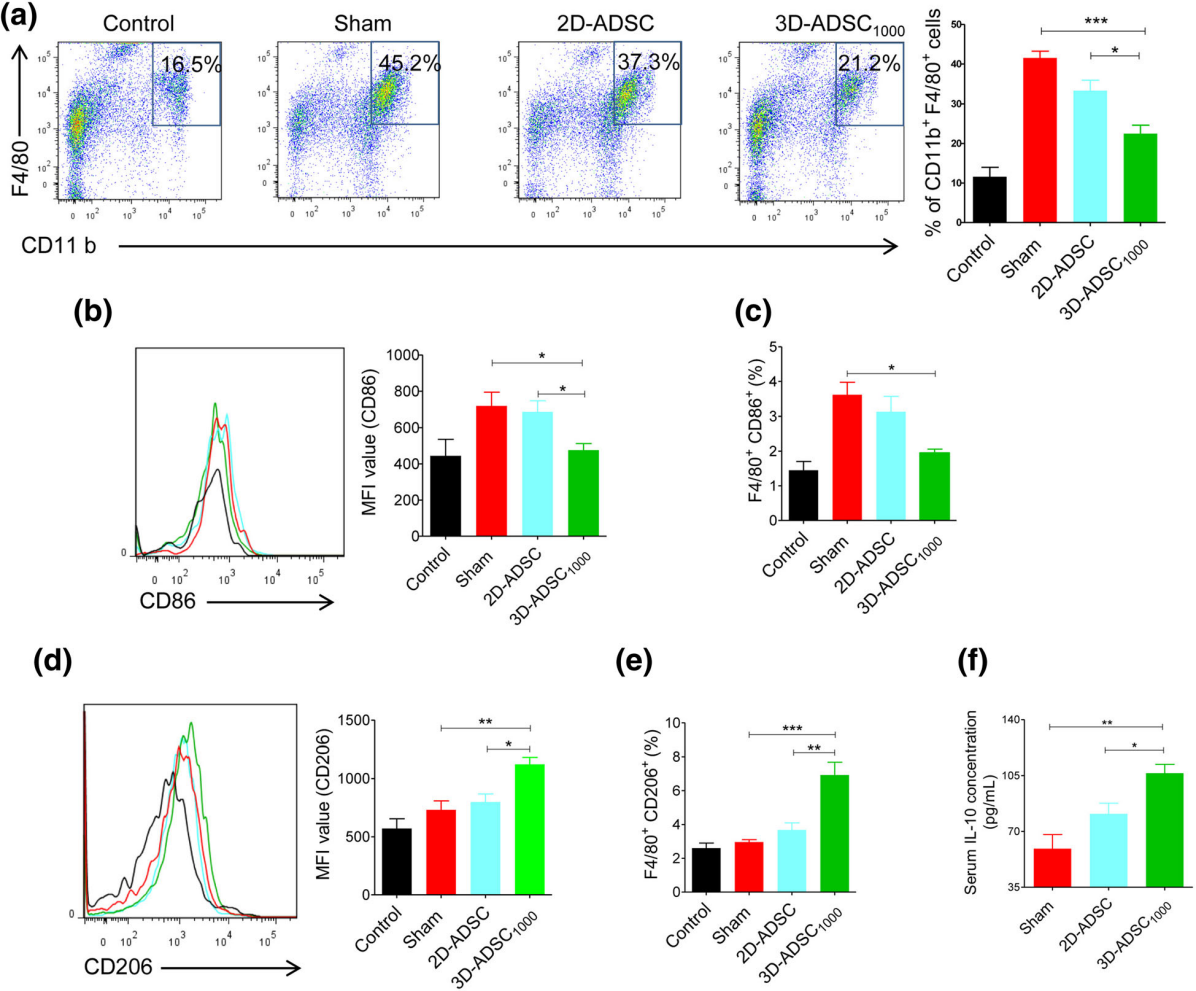
In vivo modulation of inflammatory responses by 3D-ADSC_1000_: **a** Flow cytometric analysis of surface markers (CD11b^+^F4/80^+^) for the determination of total macrophages in the liver of mice. The percentage represents CD11b^+^F4/80^+^ population, and the graph represents mean ± SEM (*n* = 4) (one-way ANOVA). **b** Expression of CD86 from F4/80^+^ population. The graph represents the mean ± SEM (*n* = 4). **c** Expression of CD206 from F4/80^+^ population. The graph represents the mean ± SEM (*n* = 4). **d** Percentage of F4/80^+^ CD86^+^ population (mean ± SEM) (*n* = 4) (one-way ANOVA). **e** Percentage of F4/80^+^ CD206^+^ population (mean ± SEM) (*n* = 4) (one-way ANOVA). **f** Serum concentration of IL-10 after 7 h of MSC transplantation (*n* = 5). *p < 0.05, **p < 0.01, and ***p < 0.001. MFI median fluorescence intensity

**Fig. 7:**
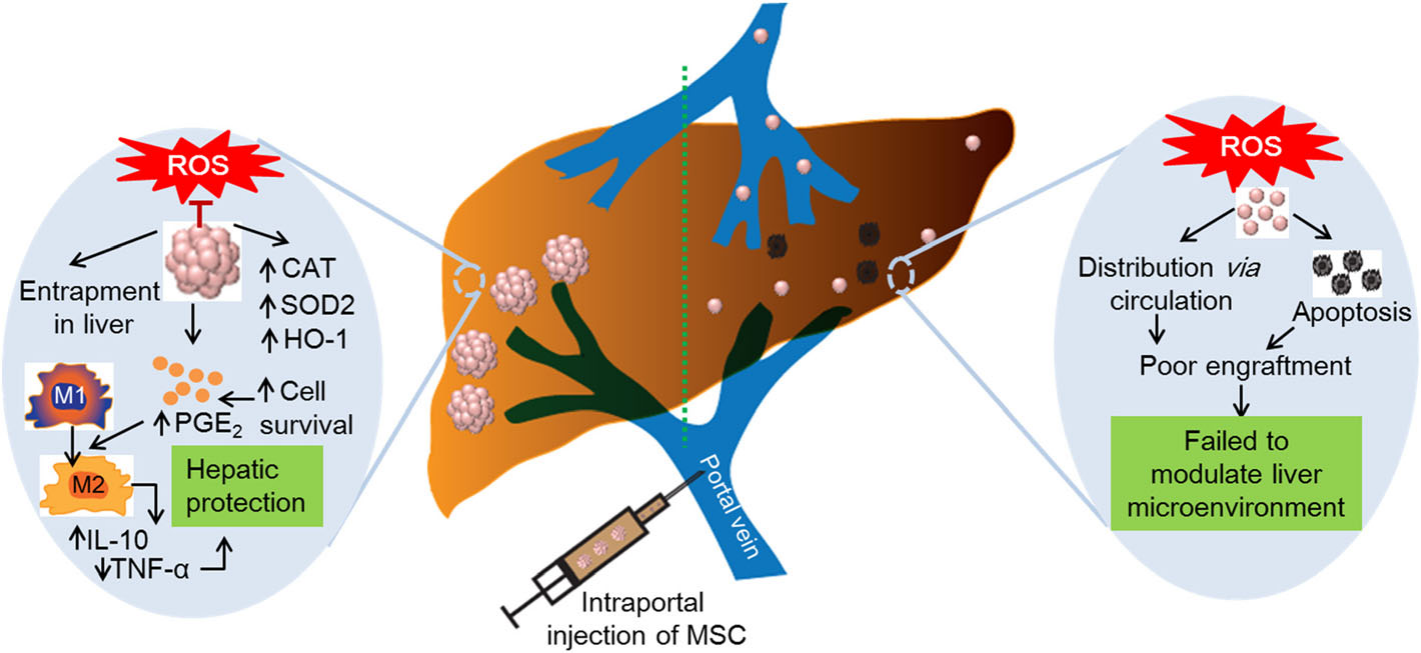
Proposed mechanism of hepatoprotection by intraportally delivered MSC spheroids. Majority of the 2D-ADSC die due to oxidative stressful environment in the inflamed liver or distribute to distant organs via circulation. This leads to a poor engraftment of MSC in the liver and failure of MSC to modulate liver microenvironment. In contrast, local delivery of 3D-ADSC via portal vein results in the engraftment of 3D-ADSC into liver sinusoids. In addition, upregulation of the antioxidant defense enzymes, such as HO-1, CAT, and SOD2, enhances survival of the 3D-ADSC against the oxidative stressful environment in the liver. This leads to a secretion of higher levels of PGE_2_ by 3D-ADSC which programs macrophage polarization from M1 to M2 phenotype. The M2 macrophages secrete higher level of IL-10 and lower level of TNF-α which results in hepatoprotection

We next investigated whether 3D-ADSC_1000_ could exert immunoregulatory efficacy in vivo. The evaluation of macrophage infiltration and polarization in the liver showed an enhanced immunomodulatory effect of 3D-ADSC_1000_ to attenuate the liver inflammation. Macrophage population (CD11b^+^F4/80^+^) was significantly upregulated in sham (*p* < 0.001), 2D-ADSC (*p* < 0.001), and 3D-ADSC_1000_ (*p* < 0.05) compared to control mice_._ However, in the 3D-ADSC_1000_-transplanted group, a significant reduction in the macrophage population was observed compared to the sham (*p* < 0.001) or 2D-ADSC groups (*p* < 0.05) (Fig. 6a). Moreover, there was a significant reduction in the expression of M1 surface marker (CD86) (Fig. 6b,c), and an upregulation of M2 surface marker (CD206) (Fig. 6d,e) among the F4/80^+^ macrophages of the 3D-ADSC_1000_-transplanted group compared to the 2D-ADSC (*p* < 0.05) or sham group (*p* < 0.001), implying the polarization of macrophage into M2 phenotype after injection of 3D-ADSC_1000_. Furthermore, an increase in serum IL-10 level in the 3D-ADSC_1000_-transplanted group supported the upregulation of M2 macrophage (Fig. 6f). These results suggest that ADSC spheroids can robustly polarize the macrophages from M1 (inflammatory) to M2 (anti-inflammatory) phenotype, presumably via elevated PGE_2_ production (Fig. 7).

## Discussion

Stem cell therapy is based on cell replacement by differentiation into desired cell types or on rearrangement of the microenvironment in a manner that promotes tissue repair [34]. MSC have been reported to be useful in FHF due to their ability to differentiate into hepatocytes [35]. Furthermore, MSC have the ability to improve the liver microenvironment and promote the regeneration of hepatocytes by regulating inflammatory cells and cytokines [14, 36]. Although MSC have been demonstrated to be beneficial in various disease conditions, their effectiveness is compromised by limited survival and engraftment after transplantation [37]. 3D-ADSC have been reported to enhance cell viability possibly due to the phosphorylation of AKT or the upregulations of hypoxia-induced survival factors and nutrient deprivation-induced factors [23, 38–40]. Recently, we have developed extracellular matrix-modified scaffolds to enhance viability and therapeutic effectiveness in inflammatory diseases [41]. In the current study, transplantation of viability-strengthened MSC spheroids via the portal vein alleviated ALF by exerting a local immunomodulatory function.

Delivery of cells via the portal vein might increase risks of venous thrombosis and embolism. In a recent study, thrombosis and infarction were more prominent after intraportal delivery of 2D-ADSC compared to the 3D-ADSC-delivered group [42], which may be due to higher expressions of thrombogenic proteins, including tissue factor (TF) and chemokine (C-C motif) ligand 2 (CCL2) in 2D-ADSC compared to that of the 3D-ADSC. Furthermore, 3D-ADSC containing 10,000 cells were reported to aggravate thrombosis and infarction in the livers [42], which indicates a necessity of control over spheroid size to protect the cells against hypoxia or nutrient deficiency after transplantation. The size-controlled MSC spheroids used for in vivo experiments in the current study survived well in a highly oxidative environment under in vitro conditions. This resembles the environment of an inflamed liver during FHF. Interestingly, no signs of hepatic thrombosis were observed in the 3D-ADSC-transplanted group.

GalN/LPS-induced FHF induces the activation of liver macrophage and the recruitment of neutrophils, monocytes, and natural killer cells in the liver to provoke inflammation [33]. Moreover, these activated immune cells and secreted inflammatory cytokines are responsible for the production of reactive oxygen/nitrogen species (ROS/RNS) [43–45] and hepatocyte death [46, 47]. Previous studies have adopted different strategies, such as treatment with antioxidants [48, 49], overexpression of anti-apoptotic Bcl-2 [50], HO-1 [51, 52], or AKT [53], and hypoxic preconditioning [54], to improve MSC survival. In the present study, we used 3D-ADSC_1000_ which had enhanced expression of Bcl-2 and HO-1 and antioxidant proteins which led the MSCs to thrive well in the oxidative stressful environment after in vivo transplantation.

MSC may actively home to tissues via leucocyte-like cell adhesion and transmigration [55]. Alternatively, they may be passively entrapped into small-diameter blood vessels [56]. We delivered 3D-ADSC_1000_ via the portal vein to ensure passive entrapment by liver sinusoids and portal triads. During systemic infusion, a large fraction of MSC are typically trapped in lung capillaries [57], and this retention often results in poor homing [58]. Thus, local delivery of stem cells to injured sites probably promotes MSC entrapment in damaged organs [57, 59]. Although the mechanisms responsible for MSC engraftment and homing at sites of injury are unknown, various homing and adhesion molecules, such as intracellular adhesion molecule-1 (ICAM-1), vascular cell adhesion molecule-1 (VCAM-1), P-selectin, integrins, and CXCR4, have been reported to be involved [60]. Long-term culture of 2D-MSC in vitro has been reported to decrease the expressions of these adhesion and homing molecules that compromise homing and engraftment efficiencies at sites of injury [61]. In a recent study, it was reported that the upregulation of CXCR4 expression in 3D aggregates promotes the adhesion of MSC spheroids with the endothelial cells [61], which would aid the localization of transplanted 3D spheroids at the site of injury. In another study, when bone marrow-derived mononuclear cell spheroids were delivered intraportally, they were found to be entrapped in the liver, whereas spheroid-derived single cells delivered intraportally drained into the systemic circulation [42].

During inflammation, high concentrations of the pro-inflammatory cytokines, such as TNF-α and IFN-γ, activate MSC to secrete soluble immunomodulatory factors, such as IDO, PGE_2_, and NO [62], which results in macrophage polarization to the anti-inflammatory phenotype (M2) and T cell switching to the regulatory phenotype [63]. Interestingly, 3D culture results in the upregulation of various pro-inflammatory cytokines, including TNF-α, IL-1α, IL-1β, and IL-8, which creates an early inflammatory microenvironment within the 3D spheroids that activates MSC in spheroids to produce immunoregulatory factors like TNF-α-stimulated gene/protein 6 (TSG_6_), stanniocalcin-1 (STC-1) [19], and PGE_2_ [20] and leads to the attenuation of inflammation or repairment of damaged tissues [64, 65]. In the present study, we observed higher COX_2_ and PGE_2_ expressions in 3D-ADSC_1000_ compared to 2D-ADSC, which resulted in more IL-10 secretion but less TNF-α secretion from macrophages co-cultured with 3D-ADSC_1000_ compared to the macrophages co-cultured with 2D-ADSC. Furthermore, an increase in M2 and a decrease in M1 macrophages in the liver suggested the better immunomodulatory role of 3D-ADSC spheroids. In our previous study, MSC were stimulated to secrete PGE_2_ by pretreating with muramyl dipeptide, which upregulates COX_2_ expression. The results reported an enhanced immunomodulatory effect in experimental colitis [66]. Similarly, IL-1β-primed MSC were reported to be more efficient at suppressing inflammation due to the upregulation of COX_2_ and PGE_2_ [67, 68]. Therefore, increased COX_2_ expression was correlated with increased anti-inflammatory effects. In the current study, 3D-ADSC effectively ameliorated ALF compared to 2D-ADSC, at least, in part, due to increased resistance to oxidative stress via the upregulation of various intracellular antioxidant mechanisms and enhanced polarization of macrophages to anti-inflammatory macrophage. Further studies should reveal the effectiveness of MSC spheroids in other inflammatory diseases.

## Conclusion

Intraportal delivery of 3D-ADSC led to the MSC engraftment into the liver and enhanced therapeutic outcomes in a mouse model of FHF. These promising results were observed due to an upregulation of antioxidant defense mechanism during the formation of 3D spheroids from monolayer-cultured ADSC. This resulted in higher cell viability after intraportal transplantation. Furthermore, the enhanced immunomodulatory activity of 3D-ADSC led to the effective polarization of the macrophage from pro- to anti-inflammatory phenotype resulting in the attenuation of inflammation in GalN/LPS-induced hepatic toxicity. Recently, Chondrosphere® has been approved in Europe for the treatment of cartilage defects in human. Several studies including ours have revealed the superiority of 3D MSC spheroids compared to the monolayer MSC. 3D MSC spheroids have a great potential to improve the therapeutic effectiveness of MSC-based therapy. However, further studies on optimization of technique for large-scale manufacturing of clinical-grade 3D spheroids and mechanism behind the improvement of MSC effect are required for clinical translation.

## Additional file

Additional file 1: **Figure S1.** Determination of lethal dose of GalN for the induction of FHF. The percentage survival of Balb/c nude mice after intraperitoneal injection of different doses of GalN (1000 mg/kg, 1500 mg/kg, 2000 mg/kg, and 3000 mg/kg) with 20 μg/kg LPS. **Figure S2.** Serum GOT and GPT level in normal mice before induction of FHF. **Figure S3.** Gross observation of the liver after 7 h of intraportal delivery of 2D-ADSC and 3D-ADSC_1000_. Massive infarction was observed throughout the liver after 2D-ADSC delivery. In contrast, minimal infarction was observed in the 3D-ADSC_1000_ delivered group (the arrows indicate the areas of the infarction). (DOCX 562 kb)

## Data Availability

Authors.

## Abbreviations

2D-ADSC: 2D-cultured adipose-derived mesenchymal stem cells
3D-ADSC: 3D spheroids of adipose-derived mesenchymal stem cells
ADSC: Adipose-derived mesenchymal stem cells
ALF: Acute liver failure
ANOVA: Analysis of variance
CAT: Catalase
CCK-8: Cell Counting Kit-8
CCL2: C-C motif chemokine ligand 2
COX_2_: Cyclooxygenase 2
CXCR4: C-X-C chemokine receptor type 4
DCFDA: 2′,7′-Dichlorofluorescin diacetate
ELISA: Enzyme-linked immunosorbent assay
FHF: Fulminant hepatic failure
GalN/LPS: d-Galactosamine/lipopolysaccharide
GOT: Glutamic oxaloacetic transaminase
GPT: Glutamic pyruvic transaminase
HO-1: Hemeoxygenase-1
ICAM-1: Intercellular Adhesion Molecule 1
IDO: Indoleamine 2,3 dioxygenase
IL-1 α: Interleukin 1 alpha
IL-10: Interleukin-10
IL-1β: Interleukin 1 beta
IL-8: Interleukin 8
MSC: Mesenchymal stem cells
PCR: Polymerase chain reaction
PGE_2_: Prostaglandin E_2_
RNS: Reactive nitrogen species
ROS: Reactive oxygen species
SIRS: Systemic inflammatory response syndrome
SOD2: Superoxide dismutase 2
STC-1: Stanniocalcin-1
TBHP: Tert-butyl hydroperoxide
TF: Tissue factor
TNF-α: Tumor necrosis factor alpha
TSG6: Tumor necrosis factor-inducible gene 6
VCAM-1: Vascular cell adhesion protein 1
