# Supplementary material for: Intraportally delivered stem cell spheroids localize in the liver and protect hepatocytes against GalN/LPS-induced fulminant hepatic toxicity
Source: Stem Cell Res Ther. 2019 Oct 16;10:230. doi: 10.1186/s13287-019-1337-3 (PMC6794806; doi:10.1186/s13287-019-1337-3)
Supplement: Supplementary file 1 — Figure S1. Determination of lethal dose of GalN for the induction of FHF. The percentage survival of Balb/c nude mice after intraperitoneal injection of different doses of GalN (1000 mg/kg, 1500 mg/kg, 2000 mg/kg, and 3000 mg/kg) with 20 μg/kg LPS. Figure S2. Serum GOT and GPT level in normal mice before induction of FHF. Figure S3. Gross observation of the liver after 7 h of intraportal delivery of 2D-ADSC and 3D-ADSC1000. Massive infarction was observed throughout the liver after 2D-ADSC delivery. In contrast, minimal infarction was observed in the 3D-ADSC1000 delivered group (the arrows indicate the areas of the infarction). (DOCX 562 kb) [file 13287_2019_1337_MOESM1_ESM.docx]

**Additional file**

**Figure S1**

**
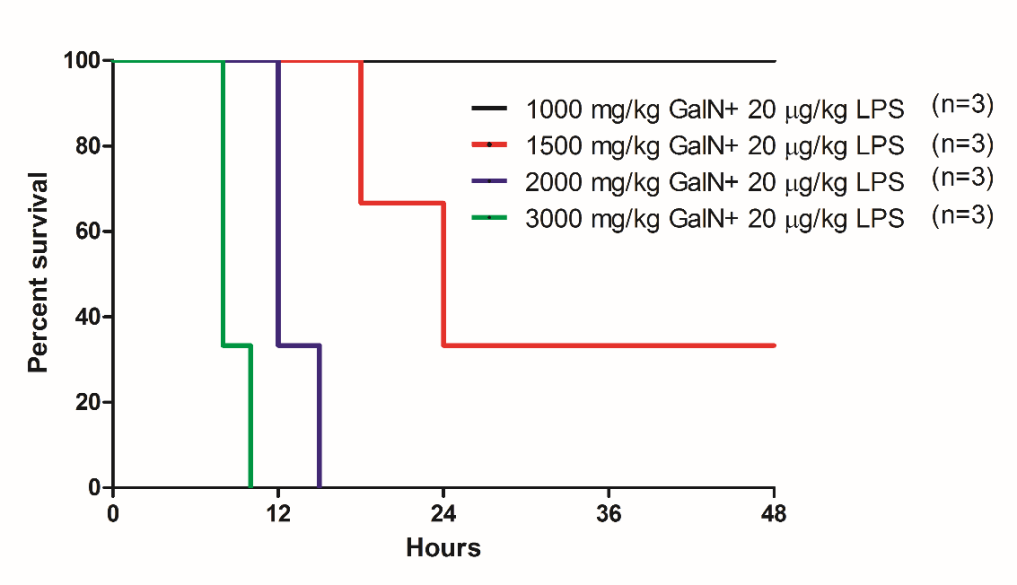
**

**Figure S1.** Determination of lethal dose of GalN for the induction of FHF**.** The percentage **s**urvival of Balb/c nude mice after intraperitoneal injection of different doses of GalN (1000 mg/kg, 1500mg/kg, 2000 mg/kg and 3000 mg/kg) with 20 µg/kg LPS.

**Figure S2**

**
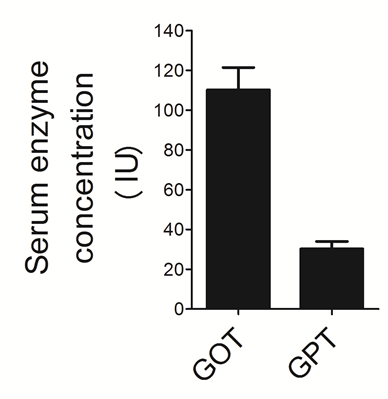
**

**Fig S2.** Serum GOT and GPT level in normal mice before induction of FHF.

**Figure S3**

**
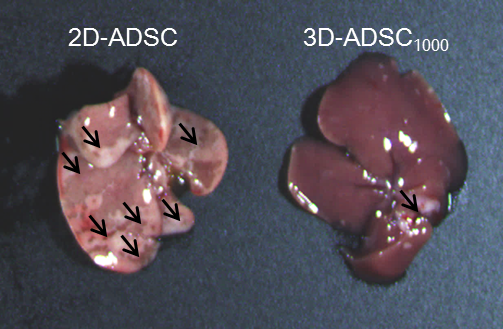
**

**Figure S3.** Gross observation of liver after 7 hours of intra-portal delivery of 2D-ADSC and 3D-ADSC_1000_. Massive infraction was observed throughout the liver after 2D-ADSC delivery. In contrast, minimal infraction was observed in 3D-ADSC_1000_ delivered group. (The arrows indicate the areas of infraction)
